# First Case of Human Anisakiosis in Greece: Acute Invasive Infection Mimicking Peritoneal Malignancy

**DOI:** 10.3390/pathogens13020149

**Published:** 2024-02-06

**Authors:** Sotirios Dinas, Anastasia Diakou, Konstantinos Vasiliadis, Serafeim C. Chaintoutis, Eleftheria Massa, George N. Konstantinou, Albion Totsi, Athanasios Xakis, Christos Papavasiliou

**Affiliations:** 1 Surgical Department, Papageorgiou General Hospital, 56429 Thessaloniki, Greece; sotiris.dinas@gmail.com (S.D.); konvasisurg@gmail.com (K.V.); almpion@gmail.com (A.T.); athanasios.xakis@hotmail.com (A.X.); hripapa@gmail.com (C.P.); 2 Laboratory of Parasitology and Parasitic Diseases, School of Veterinary Medicine, Faculty of Health Sciences, Aristotle University of Thessaloniki, 54124 Thessaloniki, Greece; 3 Diagnostic Laboratory, School of Veterinary Medicine, Faculty of Health Sciences, Aristotle University of Thessaloniki, 54627 Thessaloniki, Greece; schainto@vet.auth.gr; 4 Department of Surgical Pathology, Papageorgiou General Hospital, 56429 Thessaloniki, Greece; elma1405@gmail.com; 5 Department of Allergy and Clinical Immunology, 424 General Military Training Hospital, 56429 Thessaloniki, Greece; gnkonstantinou@gmail.com

**Keywords:** *Anisakis*, anisakidosis, anisakiasis, ectopic, intestinal anisakiasis, extra-gastrointestinal, foodborne, zoonotic, sushi, colectomy

## Abstract

Consumption of raw and mildly processed seafood, in the context of modern Western world eating trends, is recognized as a major driver for human fish-borne infections. However, these zoonoses and their unfamiliar risks remain neglected and underappreciated among European diagnosticians. In contemporary Europe anisakidosis is one of the most important fish-borne zoonoses. It is caused by ingesting the third-stage infective larvae of the nematode parasites that belong to the family Anisakidae. The case described herein, is an intestinal and ectopic form of anisakiosis (*Anisakis* spp.), causing symptoms of subacute abdomen and masquerading as an intraperitoneal malignancy. It is the first anisakidosis case reported in Greece, affecting a young patient who had been repeatedly exposed to the parasite by consuming homemade raw fish. Right hemicolectomy, omentectomy and excision of a descending colon nodule were uneventfully performed. The pathology report confirmed granulomatous tissue with eosinophilic infiltration and parasites that were morphologically and molecularly identified as *Anisakis* spp. Although challenging, acquiring an accurate diagnosis of anisakidosis can prevent unnecessary surgery, as the infection typically is self-resolving, and if treatment is deemed necessary, it can be limited to antiparasitic medication. However, in rare cases, extra-gastrointestinal migration of larvae can cause severe damage with practically unknown risks, posing a diagnostic and therapeutic dilemma. In such a clinical case scenario, surgical exploration can decisively contribute to a definitive diagnosis and early identification of intraabdominal complications necessitating surgical intervention.

## 1. Introduction

Anisakidosis is a parasitic infection caused by nematode parasites belonging to the family Anisakidae, genera *Anisakis*, *Pseudoterranova* and *Contracaecum*. Parasites of the genus *Anisakis*, i.e., *Anisakis simplex* sensu stricto and *Anisakis pegreffii* are the most frequent causative agent of human anisakiosis [1]. *Anisakis* spp. has an indirect life cycle with marine mammals, i.e., porpoises, dolphins and whales as the definitive hosts. Adult *Anisakis* spp. parasitise the gastrointestinal tract of these animals and produce eggs that are shed via the faeces into the water, where the developed larvae hatch and infect the intermediate hosts, i.e., various marine crustaceans. When consumed, the third-stage larvae (L3) in marine crustaceans are infective for the final hosts. Sea fish and cephalopods that consume infected crustaceans, can harbour the infective L3 in their tissues, serving as paratenic hosts of the parasite. In fact, the infective larvae may have several passages from smaller to bigger fish, following the trophic chain in the sea until they reach their definitive host, where they eventually develop into adult nematodes [1,2].

Humans and fish-eating terrestrial animals are accidental and usually dead-end paratenic hosts of these parasites. Thus, humans acquire the infection by ingesting raw or undercooked seafood containing the infective L3 [2], which invades the gastrointestinal mucosa causing direct gastric and intestinal damage or, rarely, extra-gastrointestinal inflammatory changes that can evolve either to acute abdomen necessitating surgical intervention or phlegmonous lesions and granulomatous changes in the peritoneum that can be misdiagnosed as tumours [3,4]. The clinical presentation of patients with anisakidosis includes abdominal pain, nausea, vomiting and allergic reactions ranging from urticaria to anaphylaxis [3,5,6]. According to some authors, allergic reactions can also occur in the presence of the parasite’s antigens, even if the parasites are consumed deactivated or dead [1,2].

The majority of human cases (over 90%) originate from countries of the Far East, particularly Japan, due to the food preparation habits in this area of the world [3]. However, an increasing number of cases diagnosed in Europe and the American continent in recent decades can be attributed to the altered eating habits in Western countries [3,5,7,8]. In the present article, a case of invasive intestinal and ectopic anisakiosis (caused by *Anisakis* spp.) is reported, in a young patient, who has been repeatedly exposed to the parasite by consuming homemade raw fish. The infection caused symptoms of subacute abdomen and mimicked intraperitoneal malignancy. Definitive diagnosis and management were accomplished by laparotomy. To the best of the authors’ knowledge, this is the first report of clinical human anisakidosis in Greece.

## 2. Case Description

A 22-year-old male patient, with an unremarkable past medical history, presented to the emergency department of Papageorgiou General Hospital, Thessaloniki, Greece, complaining of abdominal pain located in the left lower quadrant that started about 12 h before. The pain was associated with nausea and shivering, without vomiting or fever. The patient’s vital signs were normal and physical examination of the abdomen revealed tenderness in the left iliac region. An elevated white blood cell count (13.1 × 10^9^/L) and slightly elevated CRP (1.2 mg/dL, with normal value < 0.5 mg/dL) were found in the laboratory test and the patient was admitted to the surgical department for further evaluation.

**Figure 1:**
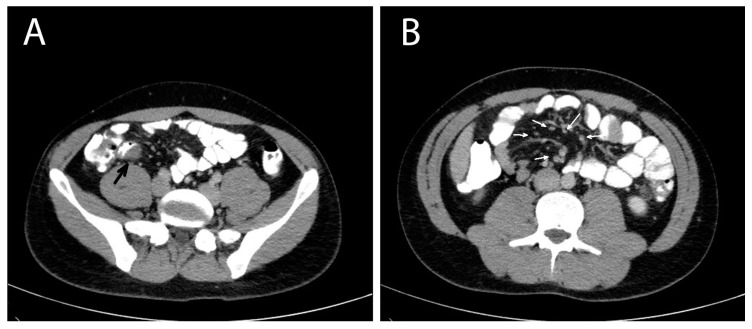
Abdomen contrast-enhanced MRI of a patient with invasive intestinal anisakiosis. (**A**): Distal ileum lesion (black arrow). (**B**): Multiple lymph nodes along ileocolic vessels (white arrows).

A contrast-enhanced computed tomography (CT) of the abdomen was performed the next day that showed multiple masses with soft tissue densities at the gastrocolic ligament (max ~1.1 cm), the left part of greater omentum (max ~1.5 cm), right paracolic gutter (max ~0.7 cm) and the left pararenal space (max ~1.7 cm), with multiple enlarged mesenteric lymph nodes along the ileocolic vessels (max ~1 cm), and at the aortic hiatus of the diaphragm (max ~1.2 cm). A thickening at the wall of the distal ileum right before the ileocolic junction was also depicted (Figure 1). The CT findings suggested a neoplastic disease and endoscopy of the upper and lower gastrointestinal tract was decided on. The oesophagogastroscopy was normal and a colonoscopy revealed a mild oedema of the mucosa of the distal ileum and a sessile polyp of the distal sigmoid. The investigation was completed with a computed tomography (CT) scan of the thorax, which was normal, and a contrast-enhanced MRI of the abdomen that depicted the same nodules and enlarged lymph nodes showed in the CT scan, in addition to a small quantity of pelvic fluid (ascites). During the investigation, the patient remained afebrile and hemodynamically stable, with, however, persistent abdominal symptoms and signs.

**Figure 2:**
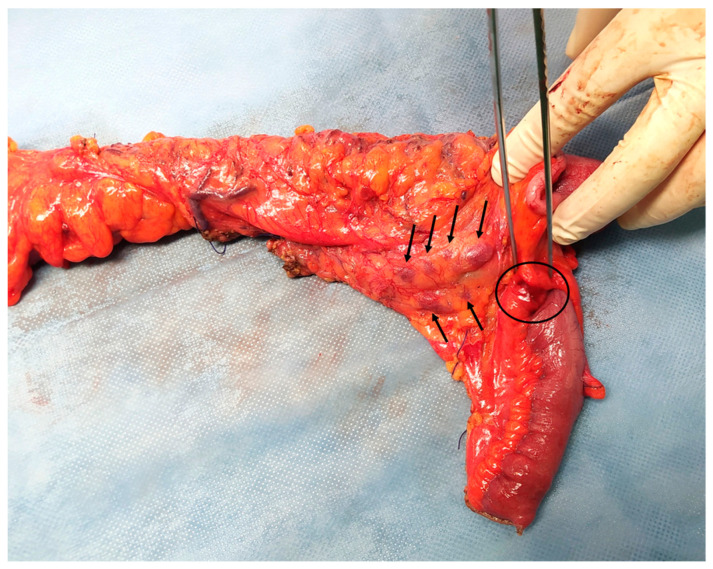
Colectomy of a patient with invasive intestinal anisakiosis. Multiple enlarged lymph nodes (arrows) and a lesion in the distal ileum (circle, also shown in Figure 1A) are visible.

Considering the abovementioned findings, the young age of the patient and the absence of a definitive diagnosis, a diagnostic laparoscopy was performed, revealing a dilated terminal ileum with localized oedema and a firm round nodule of ~1 cm in diameter in the mesenteric border of the distal ileum, about 2 cm from the ileocolic junction. Many enlarged lymph nodes were found along the ileocolic and middle colic vessels and the greater omentum. Another firm nodule was found in the mesocolic border of the descending colon approximately 2 cm in diameter (Figure 2). There were no signs of diffuse peritoneal disease. Neoplasia was suspected and the operation converted to an open laparotomy through which an extended right colectomy and an omentectomy were performed. A side-to-side ileotransverse anastomosis re-established the continuity of the gastrointestinal tract. The nodule of the descending colon was also excised. The patient had an uneventful course and was discharged on postoperative day 13.

The pathology of the surgical specimens revealed granulomatous tissue with eosinophilic infiltration at the masses at the terminal ileum, descending colon and omentum and granulomatous lymphadenitis with no sign of malignancy. Parasitosis was regarded as the most possible cause of the lesions. Accordingly, a faecal sample, blood serum and histology preparation were sent to the Laboratory of Parasitology and Parasitic Diseases, in the School of Veterinary Medicine, of the Aristotle University of Thessaloniki for further examinations. Treatment with albendazole (400 mg BID) for 28 days was initiated. This is a common scheme for the treatment of tissue parasitoses [9], thus it was selected for the present case of extended invasion by the parasites of the intestinal wall and adjacent tissues. Two weeks after the completion of antiparasitic therapy a CT scan of the abdomen confirmed no residual disease.

## 3. Parasitological, Molecular, and Serological Examinations and Results

**Figure 3:**
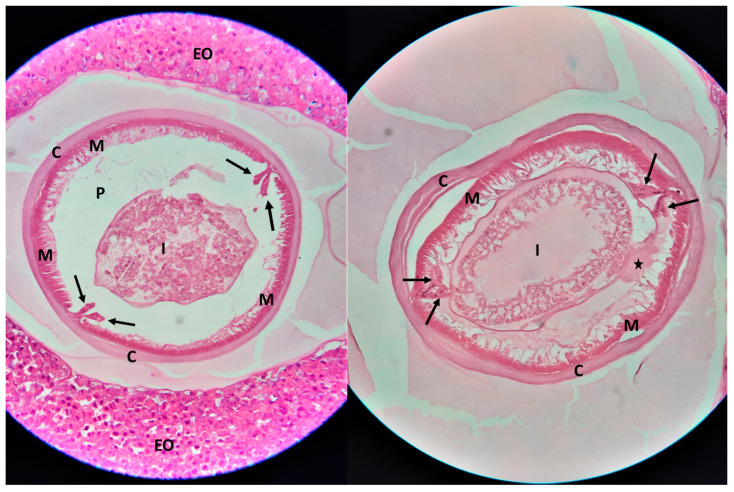
Two histological sections (hematoxylin and eosin; original magnification ×400) of *Anisakis* spp. in surgically excised granulomatous lesions of the intestine and the mesentery. The cross-section of the nematode’s body shows a smooth cuticle (C), a pseudocelomatic cavity (P), polymyarian/coelomyarian type muscle layer (M), the intestinal tract (I), prominent Y-shaped (clover-shaped) lateral chords (arrows), and the excretory organ (asterisk). The parasite is surrounded by granulomatous tissue with inflammatory cells, mainly eosinophils (EO).

At the microscopical examination of the histological samples, cross sections of a nematode parasite were observed, in the middle of granulomatous lesions. The morphological characteristics of the nematode included a smooth cuticle, polymyarian/coelomyarian type muscle layer, prominent Y-shaped (clover-shaped) lateral chords, and a structure resembling the eosinophilic excretory organ (Renett cells) (Figure 3). According to the morphological characteristics, the parasite was identified as *Anisakis* spp. [10,11].

Sections from paraffin-embedded tissue biopsy underwent DNA extraction. Paraffin was first removed via xylene deparaffinization as follows: sections were placed in a microcentrifuge tube and were mixed with 1 mL of xylene, followed by incubation at room temperature for 30 min. After centrifugation (11,000× *g*, 3 min) and discarding the supernatant, sections were subjected to a series of wash steps, using 100% and 75% ethanol, and PBS (1 mL each), respectively. All subsequent steps of the DNA extraction process were performed utilizing a commercially available DNA extraction kit (NucleoSpin Tissue; Macherey-Nagel, Düren, Germany).

The detection of the *Anisakis* spp. genome was performed via a previously published [12] genus-specific real-time PCR protocol targeting a 58 bp fragment of the internal transcriber spacer 1 (ITS1) region (positions 156–213 on *A. simplex* isolate ANE01, GenBank Acc. No. OR717507.1). The PCR reaction (25 μL) was composed of QuantiFast SYBR Green PCR Master Mix (Qiagen, Hilden, Germany), 1 μM of each of the primers (Anir18F and Anir18R), 1 μL of Template DNA and nuclease-free water. A CFX96 Touch Real-Time PCR Detection System (Bio-Rad Laboratories, Hercules, CA, USA) was used. The following cycling conditions were applied: 95 °C for 5 min (initial denaturation and Taq polymerase activation), and 40 cycles in 2 steps: (i) 95 °C for 10 sec (denaturation), and (ii) 56 °C for 30 s (annealing/extension), followed by fluorescence measurement. Subsequently, a melting curve was generated by heating the tubes from 65 °C to 90 °C in 0.2 °C increments. Fluorescence data were analysed using the CFX Maestro Software (v4.1; Bio-Rad Laboratories, Hercules, CA, USA). Real-time PCR yielded a positive result with a melting curve analysis revealing a temperature peak of approx. 80 °C, specific for the ITS1 of *Anisakis* spp. Characterization at the species level was not feasible, due to compromised DNA integrity caused by formalin fixation, which did not allow for the successful amplification of longer PCR products required for this purpose.

A serum sample collected from the patient on the day of the operation was examined by an in-house ELISA, using crude antigen of *Anisakis* spp., prepared as described before [13] from parasites isolated from fish in a previous study [14]. Specific anti-*Anisakis* IgG [0.750 optical density units (OD), with a negative control at 0.120 OD] were detected. The serological examination was repeated 2 and 5 months after the completion of treatment with albendazole, scoring negative for specific anti-*Anisakis*-antibodies.

The parasitological examination of faeces (ZnSO_4_ flotation and formalin-ether sedimentation methods) scored negative.

## 4. Discussion

The case described herein is the first clinical human anisakidosis reported in Greece. Following the confirmation of the nematode parasite as the cause of the excised nodular lesions and targeted questions directed at the patient, it was disclosed that he regularly consumed various raw fish (sushi), nearly on a daily basis. A noteworthy detail of the anamnesis was that a considerable portion of the sushi he consumed was self-prepared, with fresh fish procured from the local fish market. Indeed, in the Greek market, the prevalence of *Anisakis* infection in fish from the Aegean Sea has been documented to be between 18.8% and 98.8% [14,15].

Anisakidosis is a parasitic disease with a worldwide occurrence. The majority of cases originate from Japan (>90%), where eating raw fish, in the form of sushi and sashimi is very common [8,16]. It is estimated that approximately 20,000 new diagnoses of anisakidosis are made every year in Japanese hospitals [17] and there is a rising number of reports from all over the world. [3,18,19]. Among all countries reporting the disease, other than Japan, Spain, South Korea, and Italy have the highest number of published cases [20]. The first case of anisakidosis in Europe was reported in the Netherlands in 1960 [21], but currently, Spain has the highest load of anisakidosis incidents reported every year [22].

Cases have been described in patients from 7 months to 85 years old, with the onset of symptoms ranging from direct (gastric anisakidosis) up to 2 months (intestinal and extraintestinal anisakidosis) after the consumption of raw or undercooked seafood. In some cases, the symptoms lasted up to 10 years [5,20].

Although anisakidosis is usually self-limiting, as the parasite survives in humans only for a few days, it may occasionally present as a severe parasitosis, with implications caused by the migratory, invasive behaviour of *Anisakis* spp.-infective larvae [8,16,23]. Accordingly, anisakidosis can present as gastric, intestinal, ectopic/extraintestinal or allergic reaction which can range from mild cutaneous manifestations to severe anaphylaxis [3,5,8]. Symptoms include abdominal pain, nausea, vomiting and low-grade fever [3]. When the stomach is affected, upper gastrointestinal endoscopy can offer both diagnosis and treatment, with the removal of the parasites [3,16,24]. Intestinal and extraintestinal disease is challenging to diagnose because there are neither specific symptoms nor pathognomonic findings from common laboratory and imaging exams [3,5,25]. The parasite invades the gastrointestinal wall, causing both direct damage to the tissue and allergic reactions, with an accumulation of neutrophils and eosinophils [5]. Sometimes it can be misdiagnosed as acute intraabdominal inflammation (e.g., appendicitis, peritonitis), bowel obstruction or neoplasia. [5,23]. Indeed, the chronic form of the disease can result in granulomatous tissue formation, presenting as firm masses difficult to differentiate from neoplasm (mesenchymal tumours), radiologically and macroscopically [3,4,16,26,27]. This form of the disease carries unfamiliar risks emerging serious diagnostic and therapeutic dilemmas. In such clinical case scenario, surgical exploration can decisively contribute to a definitive diagnosis and early identification of intraabdominal complications necessitating surgical intervention [20], as in the present case.

The biochemical profile and imaging features of patients with anisakidosis are nonspecific, including leucocytosis, with or without eosinophilia, CRP elevation, gastric or bowel wall thickening, lymphadenopathy and ascites [3,5,6,28]. Imaging examinations can be more helpful but still not conclusive. Bowel wall thickening, lymphadenopathy and ascites are usually found in CT scans [3,5,6,28,29]. Ultrasound examination can be also used for the evaluation of patients, but it requires a high rate of suspicion and is inferior to CT [30,31]. Serological confirmation of the diagnosis, with the detection of antibodies against *Anisakis* spp. with ELISA is useful, especially for intestinal and ectopic disease, but is not widely available [3,16].

In the present case, neither biochemical nor radiological examinations led to a definitive diagnosis. Furthermore, the radiological report suggested a possible neoplastic cause of the disease while an endoscopic biopsy on the distal ileum lesions was not possible. Similarly, frozen section biopsies during the operation could not exclude neoplasia. Taking into account the extent of the lesions, the age of the patient and the possibility of neoplasia, in the absence of another possible diagnosis at that time, a right colectomy with a D2 lymphadenectomy and excision of the omentum and the descending colon nodule was decided. The pathology report of the specimen suggested granulomatous tissue, and because of the eosinophilic infiltration of the lesions, parasitosis was regarded as the most probable cause. Microscopic examination of the nodules of the greater omentum, revealed nematode parasites in the centre of the lesions. The morphological identification of the parasite as *Anisakis* spp., the detection of specific antibodies in the patient’s blood serum, and ultimately, the molecular confirmation of the causative agent by real-time PCR, led to the unequivocal diagnosis of intestinal and ectopic anisakiosis in the present case.

Patients usually require only conservative treatment with fluid replantation and analgesics, as the disease has a self-limiting course and parasites commonly survive in the human body only for a few days [8,16,23]. Rarely, when complications occur due to gastrointestinal wall penetration and perforation, bowel wall obstruction not amenable to conservative treatment, or neoplasia cannot be excluded, surgery is needed [23,25,32]. Ectopic and chronic forms of the disease are rare and can pose serious diagnostic and therapeutic challenges. Albendazole (400–800 mg daily for 6–21 days) has been successfully used for the treatment of intestinal anisakidosis [33,34]. In the present case, the administration of albendazole 400 mg twice daily for 28 days proved effective in clearing any remaining parasites, as after one cycle of treatment the patient had negative serological testing and no radiological signs of residual disease.

The appropriate processing of seafood is paramount to prevent human infection by parasites of the family Anisakidae. The European Food Safety Authority suggests freezing fishery products at −20 °C for at least 24 h if they are to be consumed raw or almost raw. Heating seafood above 60 °C and salting at high concentrations and for prolonged time can also kill the larvae [19]. Nevertheless, freezing and heating do not eliminate *Anisakis* spp. antigens, therefore, may not prevent allergic reactions or sensitization [35].

## 5. Conclusions

Anisakidosis is a zoonotic disease with a self-limiting course. Thus, therapeutic operation is generally unnecessary. When certain implications occur, surgery is needed. Apart from being underappreciated, it should also be considered that in extremely rare cases, extra-gastrointestinal *Anisakis* larva localisation may cause serious intra-abdominal complications with practically unknown risks, posing a diagnostic and therapeutic dilemma. Surgical exploration can be helpful in aetiological diagnosis and the early detection of intra-abdominal complications. Herein, the first case of anisakiosis in Greece is described in a 22-year-old patient, subjected to right hemicolectomy for suspected mesenchymal neoplasia with peritoneal dissemination and diffuse lymphadenopathy. Due to the nonspecific symptoms, laboratory, and radiologic findings, and the high prevalence of *Anisakis* spp. infection of fish from the Aegean Sea, it is possible that the disease is underdiagnosed.

## Data Availability

Data are contained within the article.
